# A Locked Nucleic Acid Antisense Oligonucleotide (LNA) Silences PCSK9 and Enhances LDLR Expression *In Vitro* and *In Vivo*

**DOI:** 10.1371/journal.pone.0010682

**Published:** 2010-05-17

**Authors:** Nidhi Gupta, Niels Fisker, Marie-Claude Asselin, Marie Lindholm, Christoph Rosenbohm, Henrik Ørum, Joacim Elmén, Nabil G. Seidah, Ellen Marie Straarup

**Affiliations:** 1 Laboratory of Biochemical Neuroendocrinology, Clinical Research Institute of Montreal, Montreal, Quebec, Canada; 2 Santaris Pharma A/S, Hørsholm, Denmark; Virginia Commonwealth University, United States of America

## Abstract

**Background:**

The proprotein convertase subtilisin/kexin type 9 (PCSK9) is an important factor in the etiology of familial hypercholesterolemia (FH) and is also an attractive therapeutic target to reduce low density lipoprotein (LDL) cholesterol. PCSK9 accelerates the degradation of hepatic low density lipoprotein receptor (LDLR) and low levels of hepatic PCSK9 activity are associated with reduced levels of circulating LDL-cholesterol.

**Methodology/Principal Findings:**

The present study presents the first evidence for the efficacy of a locked nucleic acid (LNA) antisense oligonucleotide (LNA ASO) that targets both human and mouse PCSK9. We employed human hepatocytes derived cell lines HepG2 and HuH7 and a pancreatic mouse β-TC3 cell line known to express high endogenous levels of PCSK9. LNA ASO efficiently reduced the mRNA and protein levels of PCSK9 with a concomitant increase in LDLR protein levels after transfection in these cells. *In vivo* efficacy of LNA ASO was further investigated in mice by tail vein intravenous administration of LNA ASO in saline solution. The level of PCSK9 mRNA was reduced by ∼60%, an effect lasting more than 16 days. Hepatic LDLR protein levels were significantly up-regulated by 2.5–3 folds for at least 8 days and ∼2 fold for 16 days. Finally, measurement of liver alanine aminotransferase (ALT) levels revealed that long term LNA ASO treatment (7 weeks) does not cause hepatotoxicity.

**Conclusion/Significance:**

LNA-mediated PCSK9 mRNA inhibition displayed potent reduction of PCSK9 in cell lines and mouse liver. Our data clearly revealed the efficacy and safety of LNA ASO in reducing PCSK9 levels, an approach that is now ready for testing in primates. The major significance and take home message of this work is the development of a novel and promising approach for human therapeutic intervention of the PCSK9 pathway and hence for reducing some of the cardiovascular risk factors associated with the metabolic syndrome.

## Introduction

In 2003, the proprotein convertase subtilisin/kexin-9 (PCSK9; a 692 amino acid protein) was discovered [1] and its high expression levels in liver and small intestine and the chromosomal localization of its gene (∼22 kb PCSK9) on 1p32.3, suggested a possible relationship to cholesterol metabolism [1]. Indeed, Abifadel *et al*. reported several gain of function variants of human PCSK9 that are associated with familial hypercholesterolemia (FH) [2]. PCSK9 is thought to accelerate the degradation of hepatic low density lipoprotein receptor (LDLR) [3]–[5] in endosomes/lysosomes [6] by direct binding of the catalytic subunit of PCSK9 to the EGF-A domain of the LDLR [7], [8]. PCSK9 over-expression in cell lines and direct intravenous injection of the protein to mice were shown to reduce LDLR levels and increase plasma LDL-cholesterol (LDL-C) [4], [5], [9], [10]. Conversely, loss of function variants of human PCSK9 or inactivation of the mouse *Pcsk9* gene, lead to decreased plasma LDL-C [11], [12], and in mice to increased hepatic LDLR protein [13], [14].

More than 50 amino acid variants of PCSK9 (*see* http://www.ucl.ac.uk/ldlr/LOVDv.1.1.0/) are known so far and some have been clearly shown to affect plasma cholesterol levels in humans [12], [15], [16]. These changes are classified as “gain-of-function” (GOF) mutations when they are associated with high levels of LDL-C, and as “loss-of-function” (LOF) mutations when associated with low LDL-C. GOF mutations result in mild to severe hypercholesterolemia. In the most severe Anglo-Saxon mutation, D374Y, total cholesterol (TC) values reach as high as 13.1 mmol/L [17], [18], which are >4 fold higher than normal. The onset of coronary artery disease (CAD) in patients with D374Y may be 10 years sooner than in heterozygous FH patients with severe LDLR mutations [19]. On the other hand, two nonsense heterozygote LOF mutations were associated with a 28% reduction of plasma LDL-C and 88% reduction in the frequency of coronary events [20]. Amazingly, complete loss of PCSK9 function has been reported in two adult women, exhibiting a strikingly low plasma level of LDL-C (∼0.4 mmol/L) [15], [21], and no immunodetectable circulating PCSK9 [15]. These findings support the hypothesis that higher levels and/or activity of plasma PCSK9 increase the levels of circulating LDL-C and TC, suggesting that long-term lowering of PCSK9 might be beneficial in reducing the incidence of CAD, and hence PCSK9 is an attractive target for treatment of dyslipidemia [22]–[24].

While the mechanism by which PCSK9 regulates LDLR degradation is not fully resolved, it seems to involve both intracellular and extracellular pathways [4], [25]. We recently reported evidence that the intracellular pathway of LDLR degradation by PCSK9 exists in various cell types and that it is distinct from the extracellular one [26].

Targeting the extracellular pathway has recently been effectively achieved in cynomolgus monkeys following intravenous injection of a specific monoclonal antibody that interferes with the PCSK9≡EGF-A interaction, with PCSK9 and LDL-C lowering effect lasting more than 2-weeks [27]. Another approach that would affect both the intra- and extracellular pathways would involve the use of antisense oligonucleotides to reduce the levels of PCSK9 transcripts. The first report employing this approach used repeated intraperitoneal injections (100 mg/kg weekly) of a water soluble chimeric 2′-O-methoxyethyl phosphorothioate 20-mer antisense oligonucleotide (ASO) in high fat diet-fed mice for 6-weeks. This resulted in ∼90% drop of PCSK9 mRNA in liver, reduced circulating total cholesterol and LDL-C by 53% and 38%, respectively, and a 2-fold increase in the level of hepatic LDLR. However, HDL-C was also reduced with 54% [28].

The second approach utilized liposome encapsulated small interfering RNAs siRNAs as injectable lipidoid nanoparticles (LNP) [29]. Notable, formulation is necessary and it is mechanistically distinct from antisense oligonucleotides. The effects of PCSK9 silencing lasted for 3-weeks after a single intravenous administration, and led to a 50–70% reduction in PCSK9 mRNA in mouse and rat liver and significantly lowered plasma LDL-C in cynomolgus monkeys, without affecting high density lipoprotein-cholesterol (HDL-C) or triglycerides [29].

The final strategy employed in this study, is to use high affinity, single stranded, unformulated, 12–16 nucleotide short locked nucleic acid (LNA) modified gap-mer antisense oligonucleotides. Such molecules have been shown to potently and safely inhibit both mRNA and miRNA targets in mouse [30] and non-human primate [31], [32] models, and several different LNA drugs are currently in clinical trials against cancer and infectious diseases.

In the present study, we present the first evidence for the efficacy of a short LNA antisense oligonucleotide (ASO) that targets both human and mouse PCSK9. We employed human hepatocytes derived cell lines HepG2 and HuH7 and a pancreatic mouse βTC3 cell line known to express high endogenous levels of PCSK9 [1]. Transfection of these cells with LNA ASO efficiently reduces the mRNA and protein levels of PCSK9 with a concomitant increase in cell surface LDLR protein levels. *In vivo* efficacy of LNA ASO was further investigated in mice by intravenous (i.v.) administration of the unformulated LNA ASO in saline solution. The level of PCSK9 mRNA was reduced by ∼60%, an effect lasting more than 16 days. The estimated ED_50_ was 9 mg/kg. Hepatic LDLR protein levels were significantly up-regulated 2.5–3 fold for at least 8 days and ∼2 fold for 16 days.

## Materials and Methods

### Ethics Statement

All experiments were performed according to the principles stated in the Danish law on animal experiments, and were approved by the Danish National Committee for Animal Experiments, Ministry of Justice, Denmark (permit number 2007/561-1292).

### Oligonucleotide design and synthesis

The LNA antisense oligonucleotide (LNA ASO) complementary to the human and mouse PCSK9 mRNA (accession # NM174936 and NM153565) was designed as described previously [33]. It is a 13-nucleotide long gapmer with the following sequence: GTctgtggaaGCG (uppercase LNA, lowercase DNA) and phosphorothioate internucleoside linkages. The oligonucleotides were synthesized using standard phosphoramidite protocols on an ÄKTA Oligopilot (GE Healthcare) at 130 µmol to 8 mmol scales employing custom made polystyrene primer supports. The DNA monomers were obtained from Proligo (Sigma-Aldrich) and the LNA monomers and solid support were produced by Santaris Pharma (commercially available from Exiqon, Denmark). After synthesis, the oligonucleotides were cleaved from the support using aqueous ammonia at 65°C overnight. The oligonucleotides were purified by ion exchange and desalted using a Millipore-membrane and were finally characterized by LC-MS (Reverse phase and ESI-MS).

### Cell cultures and transfections

Hepatic HepG2 cells (American Type Culture Collection, Manassas, VA) were maintained in complete medium consisting of EMEM (Invitrogen, Burlington, Ontario, Canada), 2 mM GlutaMAX (Invitrogen), 1×NEAA (Invitrogen), and 10% fetal bovine serum at 37°C, 5% CO_2_. HuH7 cells (a gift from Francois Jean, University of British Columbia) were routinely cultivated in Dulbecco's modified Eagle's medium plus 10% FBS. Mouse pancreatic insulinoma β-TC3 cells (a gift from Doug Hanahan, University of California San Francisco) were also maintained in Dulbecco's modified Eagle's medium with 10% FBS.

#### HepG2 transfection in 6-well plates

At the time of transfection 250 µl of a mix of 10.2 µg/ml Lipofectamine 2000 (Invitrogen) in OptiMEM I (Invitrogen) was added to each empty well in a 6-well plate and left at room temperature for 5 min. Next, a 250 µl mix of oligonucleotide at appropriate concentration and OptiMEM I was added to each well gently mixed and left at room temperature for 15 min. Finally a 1 ml suspension of 6.5×10^5^ HepG2 cells in complete media was added to each well and cells were incubated for 4 h at 37°C, 5% CO_2_ (final concentration: 1.7 µg/ml of Lipofectamine 2000, 0–25 nM of LNA oligonucleotide). After incubation the cells were washed in 2 ml OptiMEM I and added fresh complete media and incubated at 37°C, 5% CO_2_ until harvested.

#### HuH7 and β-TC3 transfections in 6-well plates

Cells were seeded one day before transfection at a density of 2.5×10^5^ cells per well in 6-well plates. At 60–70% confluence, cells were transfected with oligonucleotides diluted in sterile water at final concentrations varying from 0–25 nM. In mock control cells, water without oligonucleotide was added. At the time of transfection cells in each well were first washed in 2 ml Dulbecco's Phosphate Buffered Saline (PBS) brought to room temperature and afterwards added 1.2 ml of a 6.3 µg/ml Lipofectamine 2000 in OptiMEM I solution. The cells were left for 7 minute at room temperature. Next 300 µl of LNA oligonucleotides at varying concentrations dissolved in OptiMEM I were added and the cells were incubated for 4 h at 37°C, 5% CO2 (final concentration: 5 µg/ml of Lipofectamine 2000, 0–25 nM of LNA oligonucleotide). After incubation the cells were washed in 2 ml PBS and added fresh complete media and incubated at 37°C, 5% CO_2_ until harvested.

For total RNA extraction, cells were harvested 24–48 h post transfection and for Western blot, transfected cells were first incubated in complete media for 2 days, followed with incubation for overnight in condition media (medium without FBS). The next day, media were collected and cells lysed.

### *In vivo* study in mice

Female NMRI mice (Taconic, Denmark) were used in all studies and were fed ad libitum (diet: altromin no 1324, 4 wt % fat, Brogaarden, Denmark). The animals were subjected to a 12 h light cycle, temperature of 21±2°C and a relative humidity of 55±10%.

The oligonucleotide was dissolved in physiological saline solution, which also served as control and was administered intravenously (i.v.) in the tail vein. The mice were dosed either a single injection of 5–40 mg/kg or weekly injections of 5 mg/kg.

At sacrifice, the mice were anesthetized (70% CO_2_/30% O_2_) before blood sampling and cervical dislocation. Liver samples were collected both in RNA-later (Sigma-Aldrich, Denmark) and snap-frozen in liquid nitrogen. All experiments were performed according to the principles stated in the Danish law on animal experiments, and were approved by the Danish National Committee for Animal Experiments, Ministry of Justice, Denmark.

### RNA preparation and cDNA synthesis

Cells were washed three times with PBS and incubated with Trizol reagent (Invitrogen). Total RNA was extracted according to the manufacturer's instructions and resuspended in 20–30 µl of RNase-DNase free water. Isolated RNA integrity was electrophoretically verified by ethidium bromide staining of agarose gel and optical density (OD), with an OD260/OD280 average absorption ratio of 1.8 to 2.0. Typically, 250 ng of total RNA were used for cDNA synthesis in a total volume of 20 µl using SuperScript II reverse transcriptase, 25 µg/mL oligo(dT) 12–18 mer, 0.5 mmol/L 2′-deoxynucleoside 5′-triphosphates, and 40 U of RNaseOUT (all products from Invitrogen).

From tissue homogenates, total RNA was extracted using the RNeasy mini kit (Qiagen, Demark). cDNA of total RNA extracted from mice tissues, was synthesized by reverse transcription using random decamers, 0.5 µg total RNA, and M-MLV RT (Ambion, Denmark), according to manufacturer's instructions.

### Quantitative PCR

Quantitative PCR (QPCR) was performed as previously described [26], [34], [35]. For each cell line, one to three RNA samples were analyzed at least twice in triplicates. Specific primers were used for the simultaneous amplification of the normalizing cDNA for ribosomal protein S14 or S16, and the cDNA for PCSK9, LDLR and HMGCoAR, as reported [14], [26], [34].

For quantification of PCSK9 and LDLR mRNAs in mouse tissues, TaqMan Gene Expression Assays (PCSK9 #Mm00463738_m1, LDLR #Mm00440169_m1, Apobec #:Mm00482894_m1 and GAPDH #4352339E) and a 7500 Fast real-time PCR instrument (Applied Biosystems, Denmark) was used. Data were analyzed with the 7500 Fast System software. Quantified mRNA levels were normalized to GAPDH and presented relative to saline control.

### Western blot analysis

Cells were washed three times in phosphate-buffered saline and lysed in RIPA buffer (50 mM Tris/HCl, pH 8.0, 1% (v/v) Nonidet P40, 0.5% sodium deoxycholate, 150 mM NaCl and 0.1% (v/v) SDS) with a Complete Protease Inhibitor Mixture (Roche Applied Science, Laval, Quebec, Canada). Proteins were separated by SDS-polyacrylamide gel electrophoresis (8% gels) and blotted on HyBond nitrocellulose membranes (GE Healthcare, Baie d'Urfe, Quebec, Canada), which were blocked for 1 h in TBS-T (50 mM Tris- HCl, pH 7.5, 150 mM NaCl, 0.1% Tween 20) containing 5% non-fat dry milk. Afterward, membranes were incubated for 3 h at room temperature in 1% non-fat milk with the respective antibodies: hPCSK9 (1∶2,500), hLDLR (1∶1,000) (R&D, Minneapolis, MN) and β-Actin (1∶5,000) (Sigma, Oakville, Ontario, Canada). Appropriate horseradish peroxidase-conjugated antibodies (1∶10,000; Sigma) were used for detection with enhanced chemiluminescence using the ECL Western blotting substrate (Pierce, Nepean, Ontario, Canada).

Western blot analysis of LDLR in tissues was performed as follows: Snap frozen liver tissue samples (approximately 30 mg) were defrosted and homogenized in 300 µl T-per Tissue Protein Extraction buffer, supplemented with Halt Protease inhibitor cocktail (Pierce, Denmark). Total protein concentrations were determined with BCA protein assay kit (Pierce, Denmark) using an albumin standard according to manufacturer's protocol. 25 µg total protein from each sample was prepared in 4×LDS sample buffer (Invitrogen, Denmark) loaded on a 4–12% Bis-Tris gel (NuPAGE, Invitrogen, Denmark) and separated for 2 h at 130 V in MOPS buffer (Invitrogen, Denmark). Proteins were blotted onto a PVDF membrane using an XCell II Blot Module (Invitrogen, Denmark) according to standard protocol. The membrane was blocked in 5% skimmed milk powder in 1×PBS over night. The membrane was there after incubated overnight in a blocking solution including primary antibodies (polyclonal goat-anti-mouse-LDLR antibody 1∶1000 dilution, R&D Systems, Denmark and monoclonal Mouse-anti-tubulin antibody, 1∶2000 dilution NeoMarkers, Denmark). Subsequently the membrane was incubated for 2 h with secondary antibodies (HRP/anti-goat 1∶2000 dilution and 1∶2000 HRP/anti-mouse, Dako, Denmark). LDLR and tubulin bands were visualized using Chemiluminescence ECL+ detection kit (Amersham, Denmark), captured with a VersaDoc5000 imaging system (Bio-Rad, Denmark) and quantified using Quantity One software (Bio-Rad, Denmark). LDLR protein levels were normalized to tubulin and presented relative to saline control.

### FACS analysis

Transfected HepG2 cells were processed for flow cytometry after 72 h of transfection. Cells were washed with buffer A (1× PBS with 7.1 ml BSA −35%, and 2.0 ml glucose −250 mg/L) and then stained with a mouse anti-human primary antibody (Santa Cruz Biotechnology) for 40 min at room temperature at dilution of 1∶100. As for secondary antibody, anti-mouse conjugated with Alexa 647 was used (1∶250) for 20 minutes at room temperature. Flow cytometry analysis was performed by BD LSR Cytometer (BD Biosciences), and the data were analyzed using the CellQuest software package (BD Biosciences). For each sample, 10,000 live events were collected.

### Cytotoxicity assay

The toxicity was evaluated by colorimetric Alamar blue assay [36]. The blue colored reagent Alamar blue contains resazurin which is reduced to a pink colored resorufin by the metabolic mitochondrial activity of viable cells and can be quantified colorimetrically and fluorimetrically. For assessing the cell viability, HepG2 cells were transfected with LNA antisense oligonucleotideas described above in the transfection experiment, in a 24-well plate. After 48 h, 50 µl of Alamar blue reagent, pre-warmed at 37°C was added to each well and incubated for another 2 h. At the end of incubation 80 µl of media containing reduced Alamar blue dye was transferred to 96 well plate and read on Elisa plate reader at 570 nm. Untreated cells were taken as control with 100% viability and cells without addition of Alamar blue were used as blank. The relative cell viability (%) compared to control cells was calculated by [(Absorbance)_sample_/(Absorbance)_control_]×100.

### Statistical analysis

Statistical analysis was performed using One-Way ANOVA with Bonferroni's Multiple Comparison as post test if the data followed a Gaussian distribution. If not, the non-parametric Kruskal Wallis test with Dunn's Multiple Comparison was used as a post test. Where only two groups were compared, Student's t-test was used for the statistical analysis. P values of <0.05 were considered statistical significant. GraphPad Prism (version 4.03, GraphPad Software) was used for the statistical analysis.

## Results and Discussion

### LNA induced gene-specific silencing of PCSK9 mRNA *in vitro*

PCSK9 is primarily synthesized in liver, kidney and small intestine of vertebrates, with a major expression in liver [1]. PCSK9 down-regulates the protein levels of the LDLR by enhancement of its intracellular metabolic pathway in subcellular acidic compartments, without affecting LDLR mRNA levels [3]–[5]. Many studies have shown the correlation of significant reduction in the risk of coronary heart disease with ‘loss-of-function’ of PCSK9 [11], [20]. Furthermore, *Pcsk9* knock-out mice exhibit decreased levels of circulating LDL-C, demonstrably due to a high concentration of cell-surface hepatic LDLR [13], [14]. These data strongly suggested that inhibitors/silencers of the PCSK9-mediated degradation of LDLR would be valuable tools to control plasma LDL-cholesterol levels [22], [24]. This hypothesis justified the testing of LNA antisense oligonucleotides as a novel approach to diminish the activity of PCSK9 on LDLR degradation.

LNA ASO was transfected into HepG2 and HuH7 cells and a β-TC3 mouse insulinoma pancreatic cell line. Cells were harvested after 24 h or 48 h and total RNA was extracted. Quantitative polymerase chain reaction (QPCR) was then performed to assess the decrease in PCSK9 mRNA expression, and measure its effect on the levels of LDLR and HMG-CoA-reductase (HMGCoAR) transcripts. As control, mock transfections were done with water instead of LNA ASO.

**Figure 1:**
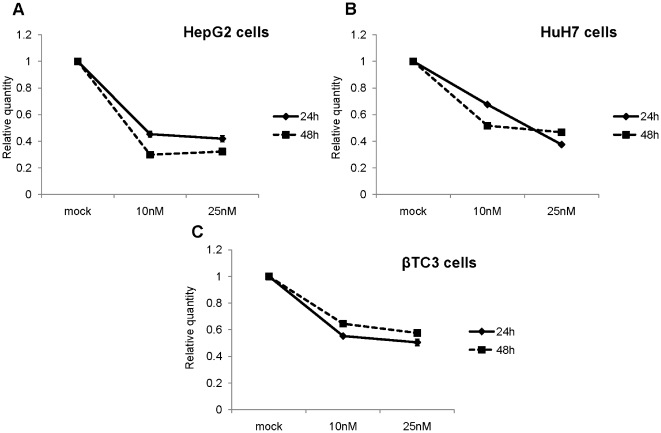
Intracellular reduction of targeted PCSK9 mRNA by LNA ASO. Two human hepatic cell lines: HepG2 and HuH7 and a mouse insulinoma β-TC3 cell line; were transfected with LNA ASO, at concentrations of 10 and 25 nM. Total RNA was extracted at two different time points- 24 h and 48 h post transfection and QPCR analysis was performed using specific primers. The levels of PCSK9 mRNA were normalized to S14 mRNA for (A) HepG2 and (B) HuH7 cells; and (C) with S16 mRNA for β-TC3 cells.

**Figure 2:**
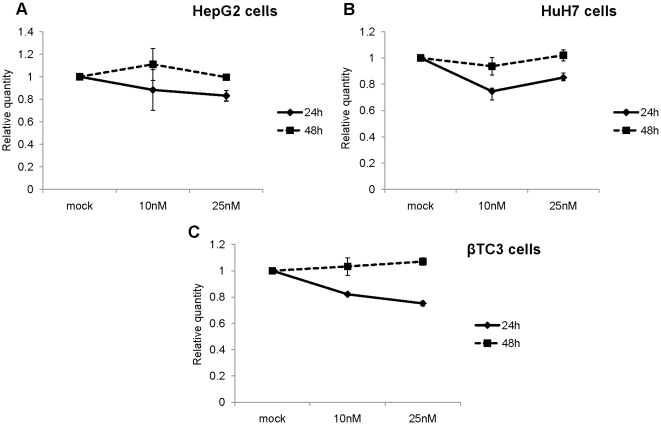
Effect of LNA ASO on mRNA expression of LDLR. Human hepatic HepG2 and HuH7 and mouse insulinoma β-TC3 cells were transfected with LNA ASO at concentrations of 10 nM and 25 nM, and the levels of LDLR mRNA analyzed by QPCR after 24 h and 48 h. The levels of LDLR mRNA were normalized to (A and B) S14 mRNA and (C) S16 mRNA for human and mouse origin, respectively.

**Figure 3:**
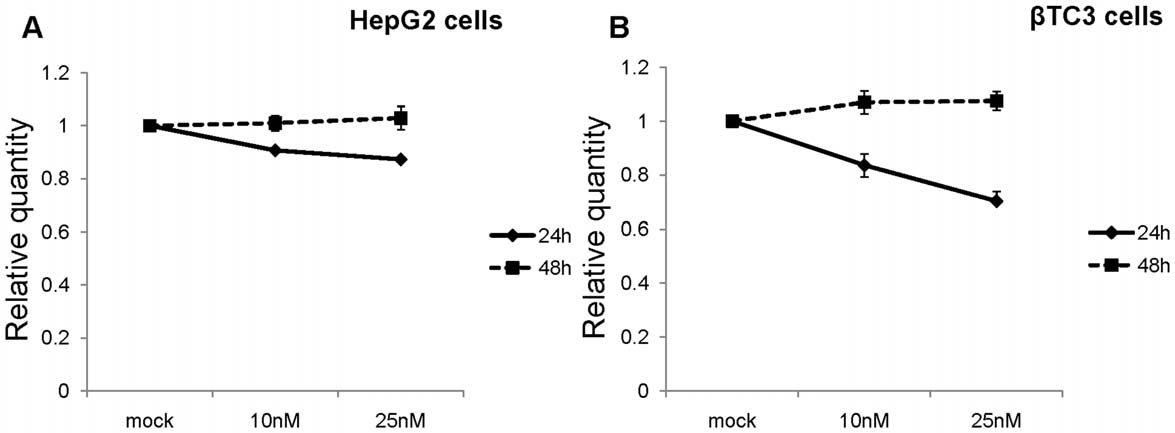
QPCR analysis of HMGCoAR mRNA. (A) HepG2 cells and (B) β-TC3 cells transfected with 10 and 25 nM LNA ASO and analyzed at 24 h and 48 h post-transfection. Human S14 and mouse S16 mRNAs were used for normalization.

As shown in Figure 1, more than 60% reduction in PCSK9 mRNA levels in human HepG2 (panel A; P<0.01), and approximately 50% in HuH7 (panel B; P<0.02) and mouse β-TC3 (panel C; P<0.05) cells were achieved after 24 h using either 10 or 25 nM LNA ASO. The same trend remained at 48 h post-transfection. We did not see any significant LNA-mediated change in the expression of LDLR mRNA in HepG2 (Figure 2A; P<0.01), HuH7 (Figure 2B; P<0.002) or in β-TC3 (Figure 2C; P<0.05) cells at the 48 h time point. Also, following 48h incubation no significant change was observed in the levels of HMGCoAR mRNA in HepG2 cells (Figure 3A; P<0.02) or in β-TC3 cells (Figure 3B; P<0.03).

The target-specificity of the LNA ASO to PCSK9 mRNA was reflected by the lack of effect on LDLR mRNA and by the use of a control untargeted LNA ASO, which showed no off-target effect on the mRNA levels of PCSK9 and LDLR in HepG2 and β-TC3 cells (Figure S1).

However, we did observe a decrease in the mRNA expression of LDLR and HMGCoAR at 24 h post-transfection (Figures 2 and 3 and S1), likely due to the down-regulation of SREBP activity in presence of lipids/sterols present in the lipofectamine reagent, as previously reported [26]. However, this effect was gone after 48h, where mRNA levels of LDLR and HMGCoAR returned to control levels.

We conclude that in the three cell lines used LNA ASO significantly inhibits the expression of human (HepG2 and HuH7 cells) and mouse (β-TC3 cells) PCSK9 mRNA, with no observable effects on the mRNA expression levels of LDLR and HMGCoAR.

### LNA ASO induced silencing of PCSK9 enhances expression of LDLR protein

**Figure 4:**
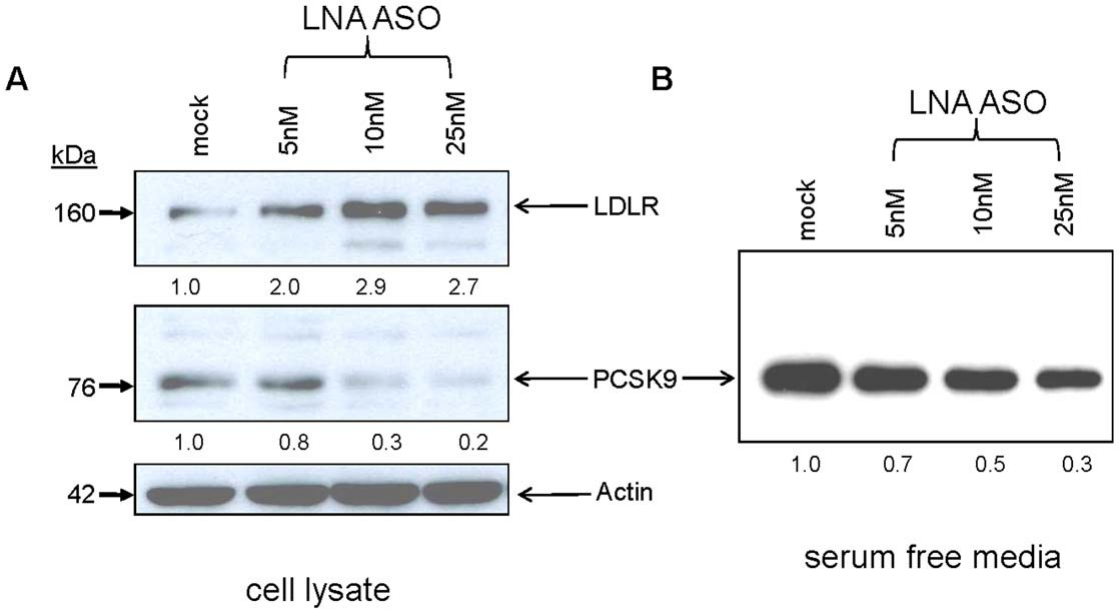
Reduction in the levels PCSK9 protein by LNA ASO is accompanied by an increase in LDLR protein expression. HepG2 cells were transfected with 5, 10 and 25 nM LNA ASO (lanes 2–4). For mock, only water was used instead of LNA solution (lane 1). (A) 72h later, cell lysates were analyzed by Western blot for endogenous levels of human PCSK9 along with LDLR protein levels. The protein β-actin was used as a loading control. (B) Western blot analysis of overnight conditioned media of LNA ASO-transfected HepG2 cells, reflecting the levels of secreted PCSK9.

Western blot analysis was done in HepG2 cells to test the effect of LNA-based silencing of PCSK9 mRNA on the protein level of PCSK9, and thus in turn on LDLR protein. For this experiment, HepG2 cells were transfected with 5, 10 and 25 nM LNA ASO, incubated for a total of 72h, and the conditioned medium analyzed was that of the last 18h. In Figure 4A, cell lysates were probed by Western blot for endogenous levels of PCSK9 and LDLR proteins in the presence and absence of LNA ASO. In panel B, The effect of the LNA ASO on the levels of secreted PCSK9 in the conditioned medium were also probed (Figure 4B). The loading control β-actin was constant in all samples. In presence of 10 and 25 nM LNA ASO we observed a decrease of ∼70–80% in endogenous cellular PCSK9 (panel A, lanes 3 and 4) and ∼50–70% in secreted PCSK9 levels (panel B, lanes 3 and 4), as compared to mock-transfected samples. This decrease in protein expression of PCSK9 was associated with a ∼3-fold increase in the levels of LDLR protein (upper panel, Figure 4A).

One of the major functions of PCSK9 is its ability to reduce the protein level of hepatic LDLR, by dragging the latter towards endosomes/lysosomes for degradation [3]–[5]. The present studies support such a role, since the LNA ASO that inhibits expression of PCSK9, results in a concomitant increase of total intracellular protein levels of the LDLR, without affecting its mRNA levels.

### FACS analysis of LDLR surface expression in HepG2 cells

To further establish the knock-down efficiency of the LNA ASO of PCSK9 mRNA, flow cytometric analysis (FACS) of HepG2 cells transfected with the LNA ASO was carried out in order to verify whether the observed increased total amounts of LDLR also reflects higher cell-surface levels.

**Figure 5:**
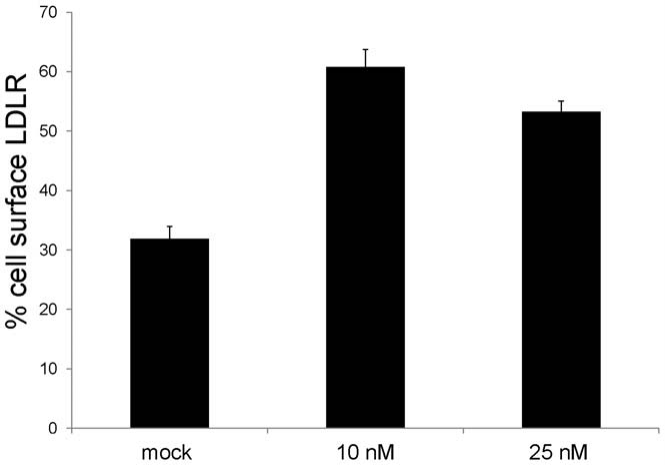
Cell surface expression of LDLR in LNA ASO treated HepG2 cells. Cells were transfected with 10 and 25 nM LNA ASO, and after 72 h FACS analysis was done to evaluate surface expression of LDLR using anti human LDLR as primary antibody and a suitable secondary antibody labeled with alexa 647.

For FACS, HepG2 cells were transfected and incubated for 72h as described in materials and methods. Cells were then probed for LDLR expression using mock cells as control. It was observed that in presence of 10 and 25 nM LNA ASO the cell surface expression of LDLR was increased by ∼50% as compared to control (Figure 5).

The FACS results revealed that the significant (P<0.01) knock-down of PCSK9 mRNA levels associated with the presence of the LNA ASO, resulting in the reduction of PCSK9 protein amounts, also leads to a 1.5-fold increase in the cell surface protein expression of the LDLR.

### Cytotoxicity of LNA ASO

**Figure 6:**
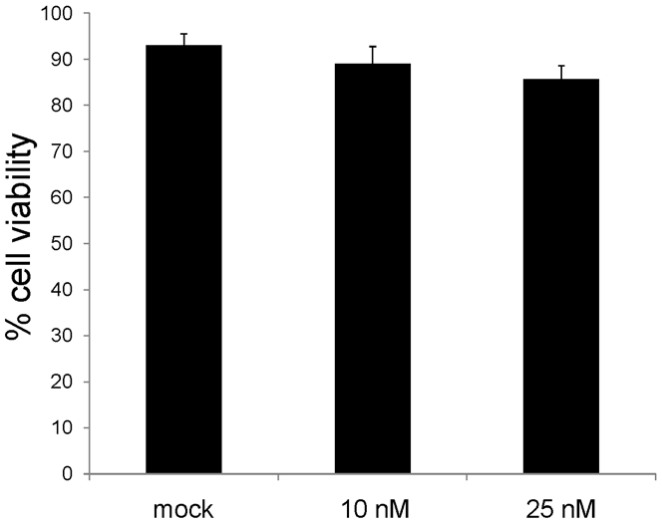
Cell viability measurement. HepG2 cells were transfected for 4h with water for mock or 10 and 25 nM LNA ASO using Lipofectamine 2000 and incubated for 48h. The cells were then treated with Alamar blue reagent and analyzed on an Elisa plate reader for cell viability.

To assess the potential cytotoxicity of the LNA ASO used in this study, cell viability was determined. Cell confluency, a qualitative measure of cell viability based upon cell coverage on the well surface, as judged by microscopy, indicated no visible toxicity at the concentrations of LNA used for transfection. To obtain a more quantitative measure of cell viability, we assayed cell metabolism activity using Alamar blue [36], [37]. LNA ASO was found to be almost non toxic, with more than 85% of the cells being viable after 48h of incubation with 10 and 25 nM LNA ASO (Figure 6).

### *In vivo* effect and dosing

**Figure 7:**
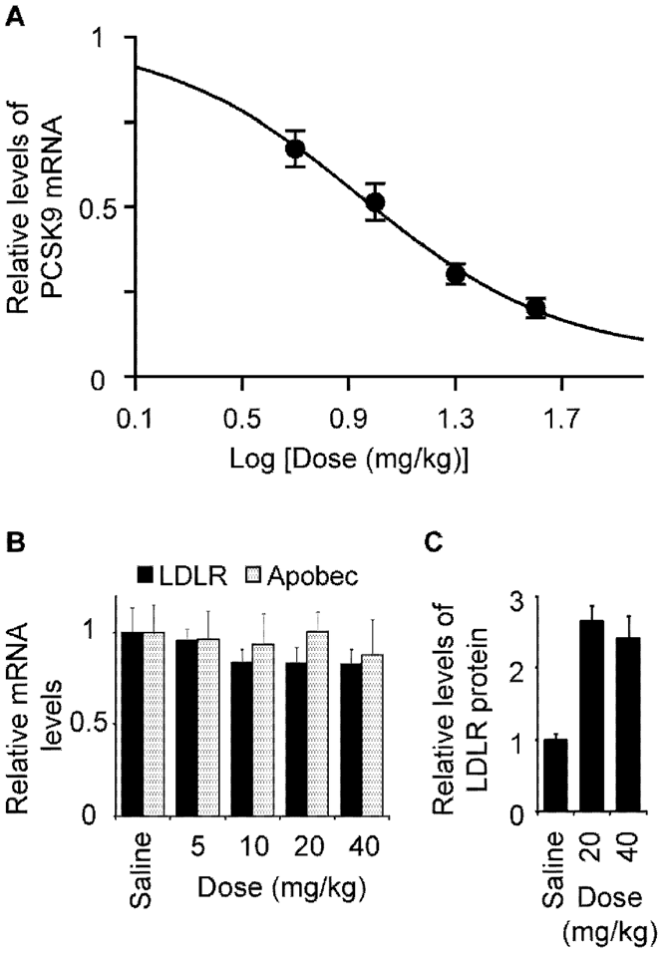
Dose-dependent PCSK9 mRNA response in mice after a single i.v. injection at 5–40 mg/kg. (**A**) Dose-response curve of PCSK9 mRNA levels in the liver (mean and SEM, n = 5), where the ED50 estimation is 9 mg/kg and maximum effect 95% reduction of the PCSK9 mRNA. (**B**) There is no change in mRNA levels of LDLR or Apobec. (**C**) LDLR protein is up-regulated 2–3 fold in the treated animals in the high dose groups. (Mean and SEM, n = 4–5).

In order to determine potency and efficacy of the 13-mer the LNA ASO *in vivo*, female NMRI mice were given a single i.v. injection of 5–40 mg/kg, sacrificed 24h post injection. Hepatic PCSK9 mRNA expression was found to be significantly reduced by the LNA ASO compared to saline (P<0.01 for all dose levels) dose-dependently and as expected with no effect on LDLR mRNA (Figures 7A and 7B). However, a concomitant increase in the level of hepatic LDLR protein by 2–3 fold was observed in the high dose groups (20 and 40 mg/kg) compared to the control treated with saline (P<0.01 for both doses; Figure 7C). The estimated ED_50_ was ∼9 mg/kg and the estimated maximum response approached 95% reduction of PCSK9 mRNA (GraphPad Prism, non-linear fit, R^2^ = 0.81; Figure 7A). The mechanistic connection was confirmed in mice and the reduction of PCSK9 mRNA resulted in an increase in LDLR protein but not LDLR mRNA levels, as also seen *in vitro* (Figure 4).

It has previously been described [28] that knockdown of PCSK9 mRNA up-regulated the apoB editing enzyme Apobec-1 by 2–3 fold. In the present study the mRNA levels of the LDLR and Apobec-1 were not influenced by treatment with the LNA ASO at any of the dose levels (Figure 7B), in agreement with the results observed in *Pcsk9* knockout mice [14]. Finally, there were no elevations in hepatic alanine aminotransferase (ALT), indicating no immediate hepatocellular injury by the LNA ASO (*data not shown*).

### Duration of action *in vivo*

**Figure 8:**
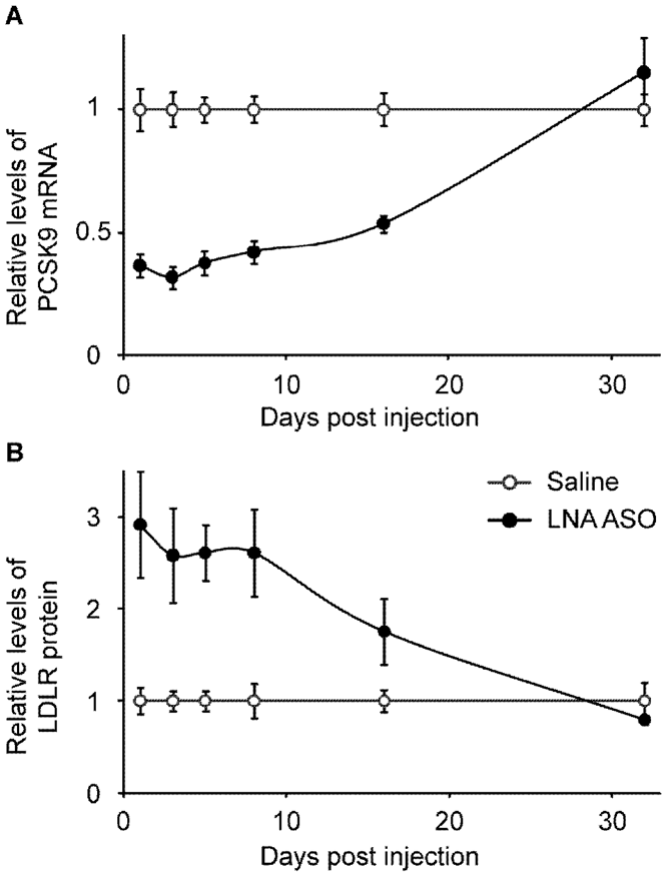
Duration of action after a single *i.v.* injection in mice. A single injection of 20 mg/kg LNA ASO generated a significant reduction of PCSK9 mRNA lasting for more than two weeks (**A**), with concomitant increase in LDLR protein (**B**). Levels were normalized at day 32. (Mean and SEM, n = 4–5).

With the objective to determine the duration of action, mice were treated with a single i.v. injection of 20 mg/kg and sacrificed at post injection days 1, 3, 5, 8, 16 and 32. The livers were analyzed for PCSK9 mRNA and LDLR protein (Figures 8A and 8B). At 24h post injection the PCSK9 mRNA was reduced by ∼60% (Figure 8A), an effect that was normalized after 32 days. The reduction was significant up to day 16 (P<0.001). The reduction in PCSK9 mRNA levels resulted in an immediate and significant up-regulation by 2.5–3 fold of the hepatic LDLR protein, an effect that lasted for at least 8 days (P<0.05) and was no significantly increased at day 16 (Figure 8).

### Effect of repeated low dose *in vivo*

**Figure 9:**
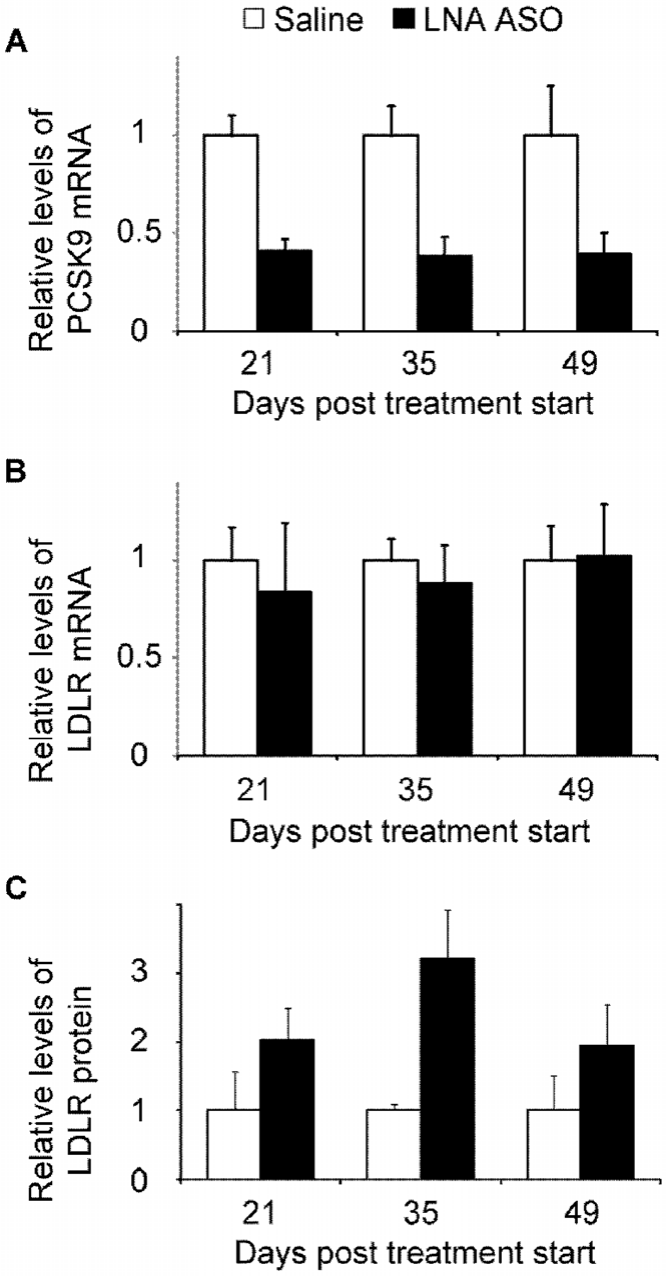
Repeated dosing in mice. Weekly repeated dosing at 5 mg/kg of the LNA ASO led to a stable reduction of PCSK9 mRNA (**A**), unchanged levels of LDLR mRNA (**B**) and 2–3 fold up-regulation of LDLR protein levels (**C**) (white bars: Saline, black bars: LNA ASO) (Mean and SD, n = 4–5).

The data for the dose-response and duration of action studies indicated that dosing 5–10 mg/kg once weekly would be sufficient to give a continuous reduction in hepatic PCSK9 mRNA expression followed by an up-regulation of hepatic LDLR protein. To validate this hypothesis and the therapeutic potential of the LNA ASO, a multiple dose study with weekly dosing of 5 mg/kg of the 13-mer LNA antisense oligonucleotide was conducted. Mice were administered i.v. 5 mg/kg/week, sacrificed on days 21, 35 and 49 (each time point occurring one week after the last dose), the livers were analyzed for PCSK9 mRNA expression and LDLR protein levels (Figures 9A and 9C). At this dose, PCSK9 mRNA had reached a significant steady reduction of ∼60% below saline treated animals (P<0.005). While LDLR mRNA levels remained constant, LDLR protein was up-regulated 2–3 fold on days 21, 35 (P<0.05) and 49 (P = 0.052) (Figures 9B and 9C).

Finally, after seven weekly doses and one week after the last dose, there was no effect on ALT activity (*data not shown*), emphasizing the lack of hepatocellular damage even after long term administration of LNA ASO.

PCSK9 down-regulates the expression of LDLR and thus directly participates in the aetiology of the hypercholesterolemia phenotype. Short, single stranded, unformulated LNA oligonucleotides hold significant promise as drugs for potent, safe and selective modulation of therapeutic interesting mRNA and miRNA targets. In the present work, we have therefore for the first time used an LNA modified antisense oligonucleotide in order to lower PCSK9 gene expression, and thus inhibit the intracellular as well as extracellular pathways of LDLR degradation. We have also shown that the LNA ASO did not affect the mRNA levels of LDLR. Taken together, this supports the specificity of the LNA ASO towards PCSK9. The use of the LNA ASO has thus reduced the expression of mRNA levels of PCSK9 and this in turn has enhanced the intracellular and surface expression levels of LDLR protein in cell lines being studied.

Both *in vitro* and *in vivo* studies presented in this work support the concept of the use of antisense LNA oligonucleotides for cholesterol lowering therapy. We are confident that LNA ASO will open the way towards future animal and clinical trials and show the therapeutic usefulness of using this PCSK9 silencing approach, as it has been for other targets.

## Supporting Information

Figure S1 QPCR analysis of PCSK9 and LDLR mRNAs in HepG2 cells transfected by an off-target LNA ASO. HepG2 cells were transfected with 10 and 25 nM of a non-target control LNA ASO, and mRNA levels of PCSK9 and LDLR in cell lysates were then measured by QPCR at 24 or 48h post-transfection. The levels of mRNAs were normalized to human S14 mRNA.
(0.02 MB TIF)
